# Association Between Environmental Factors and Asthma Using Mendelian Randomization: Increased Effect of Body Mass Index on Adult-Onset Moderate-to-Severe Asthma Subtypes

**DOI:** 10.3389/fgene.2021.639905

**Published:** 2021-05-20

**Authors:** Tae-Woong Ha, Hae-Un Jung, Dong Jun Kim, Eun Ju Baek, Won Jun Lee, Ji Eun Lim, Han Kyul Kim, Ji-One Kang, Bermseok Oh

**Affiliations:** ^1^Department of Biochemistry and Molecular Biology, School of Medicine, Kyung Hee University, Seoul, South Korea; ^2^Department of Biomedical Science, Graduate School, Kyung Hee University, Seoul, South Korea

**Keywords:** asthma, environmental factors, body mass index, mendelian randomization, moderate-to-severe asthma

## Abstract

Although asthma is one of the most common chronic diseases throughout all age groups, its etiology remains unknown, primarily due to its heterogeneous characteristics. We examined the causal effects of various environmental factors on asthma using Mendelian randomization and determined whether the susceptibility to asthma due to the causal effect of a risk factor differs between asthma subtypes, based on age of onset, severity of asthma, and sex. We performed Mendelian randomization analyses (inverse variance weighted, weighted median, and generalized summary-data-based Mendelian randomization) using UK Biobank data to estimate the causal effects of 69 environmental factors on asthma. Additional sensitivity analyses (MR-Egger regression, Cochran’s Q test, clumping, and reverse Mendelian randomization) were performed to ensure minimal or no pleiotropy. For confirmation, two-sample setting analyses were replicated using BMI SNPs that had been reported by a meta-genome-wide association study in Japanese and European (GIANT) populations and a genome-wide association study in control individuals from the UK Biobank. We found that BMI causally affects the development of asthma and that the adult-onset moderate-to-severe asthma subtype is the most susceptible to causal inference by BMI. Further, it is likely that the female subtype is more susceptible to BMI than males among adult asthma cases. Our findings provide evidence that obesity is a considerable risk factor in asthma patients, particularly in adult-onset moderate-to-severe asthma cases, and that weight loss is beneficial for reducing the burden of asthma.

## Introduction

Asthma is one of the most common chronic diseases, affecting children and adults; yet, much remains to be determined with regard to its etiology (Subbarao et al., 2009). Approximately 300 million persons have been afflicted by asthma worldwide (Dharmage et al., 2019), and it is estimated that 250,000 people die prematurely each year due to asthma (Bousquet et al., 2010). If current trends continue, roughly 100 million more persons will develop asthma by 2025 (Dharmage et al., 2019). However, asthma has considerable heterogeneity, including its pathophysiological mechanisms, environmental exposure, comorbidities, underlying disease severity, age of onset, medical accessibility, psychological factors, and medical responsiveness (Bousquet et al., 2010). For this reason, the identification of causal risk factors in a specific asthma case has been a challenging task.

Asthma is characterized by variable narrowing of the airway due to the interaction of airway inflammation and remodeling. Several studies have classified asthma by various clinical criteria, such as age of onset (early onset, late onset) (Ferreira et al., 2019), disease severity (mild, moderate-to-severe) (Shrine et al., 2019), and allergy (atopic, non-atopic) (Zhu et al., 2020). In addition, a gender-specific asthma cluster analysis has reported heterogeneous characteristics of asthma between sexes (Hsiao et al., 2019). T-helper type 2 (Th2) inflammation is regarded as the central molecular mechanism of asthma, and based on Th2 profiles, asthma is classified as Th2-high and Th2-low (Woodruff et al., 2009; Sterk and Lutter, 2014).

The severe form of asthma is aggravated by marked thickening of the airway walls and widespread inflammation (Busse et al., 2000). These changes can reduce lung function and cause the airway to be narrowed rapidly due to smooth muscle contraction (Busse et al., 2000). Thus, individuals with severe asthma present with symptoms that are distinct from those in mild asthma patients, including debilitating lung function, frequent exacerbation of asthma, and increased hospital admissions despite significant use of medicines (corticosteroids) (Shrine et al., 2019). Classification of asthma by phenotype provides a foundation on which to understand disease causality and develop management approaches that improve the control of asthma while avoiding adverse effects and decreasing the risk of serious outcomes (Moore et al., 2010).

Many environmental factors, such as BMI (Taylor et al., 2008), smoking (Hedman et al., 2011), physical inactivity (Cordova et al., 2017; Vlashki et al., 2018), and dietary habits (Protudjer et al., 2012), have been epidemiologically and clinically related to the occurrence or severity of asthma. But, observational studies encounter limitations in determining a causal link, due to their various potential confounding factors (Sun et al., 2020). Randomized controlled trials (RCTs), if appropriately conducted, are the standard for determining causal inferences in health sciences. However, RCTs are expensive and time-consuming, involving thousands of patients, some of whom might experience unwanted side effects of drugs (Roberts, 2018).

With some similarities to RCTs, Mendelian randomization (MR) is an alternative approach for establishing evidence of causal relationships for which RCTs are practically unavailable (Smith and Ebrahim, 2003). MR is a method from genetic epidemiology that uses randomly inherited genetic instruments [single-nucleotide polymorphisms (SNPs)] that are robustly associated with a risk factor as proxies for environmental exposure to assess causal inferences for the effects of exposure on an outcome (Smith and Ebrahim, 2003; Rosoff et al., 2019). The increasing availability of summary-level data from genome-wide association studies (GWASs) in the public domain allows MR to make inferences on causality by integrating summary-level GWAS data from various studies (Bowden et al., 2016).

The aim of our study was to estimate causal inferences of environmental factors on asthma using MR and to examine the differences in the susceptibility to asthma with regard to the causal effect of an environmental factor between asthma subtypes, classified by the age of onset, disease severity, and sex.

## Materials and Methods

### Study Population and Design

The UK Biobank is a population-based cohort that recruited over 487,409 individuals aged 40–69 years from 2006–2010 (Collins, 2012). For quality control of the samples, we used the following filter parameters of the Neale lab^1^ : PCA calculation filter for selection of unrelated samples; sex chromosome filter for removal of aneuploidy; filter of principal components (PCs) for European sample selection to determine British ancestry; and filters for selection of self-reported ‘white British,’ ‘Irish,’ and ‘White.’ The UK Biobank has been granted ethical approval to collect data on participants by the North West Multicentre Research Ethics Committee, the National Information Governance Board for Health & Social Care, and the Community Health Index Advisory Group.

Asthma cases (*n* = 35,926) were determined as those that had been diagnosed with asthma by a doctor and had checked for age of onset. Participants who had been diagnosed with chronic obstructive pulmonary disease (COPD) were excluded. Controls (*n* = 227,924) were defined those who had not been diagnosed with asthma, rhinitis, eczema, allergy, or emphysema/chronic bronchitis. In addition, those who had diagnostic records of hay fever, allergic rhinitis, emphysema, chronic bronchitis, or COPD and those who had J40-47 records in the ICD 10 codes (the 10th revision of the International Statistical Classification of Diseases and Related Health Problems) were excluded (Supplementary Material and Supplementary Figure 1).

For the study of specific asthma subtypes, cases were divided into 4 groups by the age of onset and disease severity: child-onset mild (CM, *n* = 9,758), child-onset moderate-to-severe (CM-S, *n* = 1,875), adult-onset mild (AM, *n* = 19,415), and adult-onset moderate-to-severe (AM-S, *n* = 4,878). Individuals with an age of onset before 19 years were defined as child-onset asthma cases (Ferreira et al., 2019), and those with an age of onset after 20 years were considered adult-onset asthma. Moderate-to-severe asthma cases were selected from individuals for whom, in addition to the conditions above, medication information was available and the British Thoracic Society (BTS) stage 3-5 criteria were met, as described (Shrine et al., 2019). Asthma cases that did not satisfy these conditions were classified as mild asthma.

To determine whether gender differences had causal effects, adult-onset asthma cases and controls were separated by gender: female adult-onset mild (FAM, *n* = 12,208), female adult-onset moderate-to-severe (FAM-S, *n* = 3075), male adult-onset mild (MAM, *n* = 7207), male adult-onset moderate-to-severe (MAM-S, *n* = 1803), female control (FC, *n* = 119,515), and male control (MC, *n* = 108,409).

### Genetic Instrumental Variants for Environmental Factors and Asthma

We studied 93 environmental factors in 18 categories that were associated with asthma (Supplementary Table 1, references attached). Association test was performed between asthma and each of the 93 factors by logistic linear regression in R, with adjustments for age, sex, and each environmental factor. As a result, 69 environmental factors were associated with asthma [*P* < 5.38E-04 (0.05/93)] (Supplementary Table 2). For each of the 69 environmental traits, we extracted SNPs that were significantly associated with each factor (e.g., SNP-environmental factor) (*P* < 5E-08) using genome-wide summary statistics, provided by the Neale lab UK Biobank GWAS summary data (Supplementary Table 3)^2^. The SNPs-environmental factor were subject to clumping (*r*^2^ > 0.05, 1-Mbp boundary distance) using FUMA (Watanabe et al., 2017) to ensure the independence of environmental factor-associated loci. As an additional quality control of SNPs for Mendelian randomization, we removed strand-ambiguous SNPs (e.g., A/T and C/G, MAF > 0.42) (Tang et al., 2020) and SNPs in the MHC region (chromosome 6:25-34M) due to their strong pleotropic effects (Supplementary Figure 2 and Supplementary Table 4; Zhu et al., 2020).

For asthma, we performed a genome-wide association analysis using UK Biobank data. Genotyping imputation was performed using the UK10K Project and 1000 Genome Project Phase 3 reference panels (UK10K Consortium, Walter et al., 2015; Genomes Project et al., 2015). General quality control procedures for exclusion (*P* for Hardy-Weinberg equilibrium test <1E-06, missing genotype call rate >0.05, minor allele frequency <0.01) were applied to 7,402,791 SNPs. In total, 5,664,578 SNPs were retained for further analysis. A GWAS for asthma was performed using PLINK 1.9, with adjustments for age, sex, genetic array, and 10PCs. A list of independent asthma-associated loci (e.g., asthma SNPs) were determined by clumping (*P* < 5E-08, *r*^2^ > 0.05, 1-Mbp boundary distance), and SNPs that were strand-ambiguous and in the MHC region were excluded. The resulting quantile-quantile (QQ) plot and Manhattan plot are shown in Supplementary Figure 3. Thus, 142 SNPs were selected for genetic instruments of asthma (Supplementary Table 5).

For the two-sample MR setting of BMI → asthma, we extracted summary association statistics for the 158 genome-wide significant SNPs (*P* < 5E-08) that were associated with BMI in a trans-ethnic meta-GWAS of 173,430 Japanese subjects (the BioBank Japan project, the Japan Public Health Center-based Prospective Study, and the Tohoku Medical Megabank Project) and 339,224 Europeans (the GIANT consortium) (total *N*_max_ = 480,438) (Akiyama et al., 2017). Of 158 BMI SNPs, we removed SNPs with palindromes (e.g., A/T and C/G) (Tang et al., 2020) and SNPs in the MHC region (chromosome 6:25-34M) in the MR analyses (Zhu et al., 2020), retaining 149 SNPs.

To avoid the biases of one-sample settings, such as reverse causality and overfitting, we performed a GWAS for BMI only in controls (*n* = 227,924) from the UK Biobank data. The resulting genome-wide significant BMI SNPs (*P* < 5E-08) were subject to clumping using FUMA (Watanabe et al., 2017). The 170 independent BMI SNPs were further subject to the removal of SNPs with strand ambiguity, SNPs in the MHC region, and SNPs that were associated with asthma, leaving 159 SNPs (Supplementary Table 10). The resulting quantile-quantile (QQ) and Manhattan plots are shown in Supplementary Figure 4.

### Mendelian Randomization

To assess the causal relationship between environmental factors and asthma, we applied 3 methods: inverse variance weighted (IVW) random effects model (Burgess et al., 2013), weighted median regression (Bowden et al., 2016; Censin et al., 2017), and generalized summary-data-based Mendelian randomization (GSMR) (Zhu et al., 2018). The causal effect estimate by IVW is liable to be biased if any SNP exhibits horizontal pleiotropy. As a complementary method to reduce heterogeneity, we performed GSMR, in which genetic variants were pruned at a high threshold of *r*^2^ < 0.05 (Pasman et al., 2019) and filtered for pleiotropic effects on exposure and outcome [Heterogeneity In Dependent Instrument (HEIDI) filtering] (Zhu et al., 2018). The weighted median method provides an unbiased estimate of the causal effect even when up to 50% of the information comes from invalid genetic variants (Bowden et al., 2016; Censin et al., 2017).

To ensure minimal or no pleiotropy in our results, we performed additional sensitivity analyses. First, we estimated the intercept by MR-Egger regression, with an intercept that differs significantly from 0 (*P* < 0.05) as an indication of residual heterogeneity due to directional pleiotropy (Bowden et al., 2015). Next, we evaluated the residual heterogeneity using Cochran’s Q statistic, with significant heterogeneity (*P* < 0.05) due to horizontal pleiotropy. Then, we removed SNPs with any evidence of pleiotropy by clumping both environmental factor and asthma SNPs (*r*^2^ > 0.05, 1-Mbp boundary distance) and excluded SNPs that were potentially associated with asthma (*P* < 0.05/number of environmental factor SNPs) for forward MR and SNPs that were linked to environmental factors (*P* < 0.05/number of asthma SNPs) for reverse MR and then repeated the MR analyses. Finally, only unidirectional causal effects were determined by performing the reverse MR of asthma → environmental factor (*P* < 5E-08).

The estimates from the IVW, weighted median, and GSMR were defined as causal effects only when meeting significance after Bonferroni correction for multiple tests as the threshold for the true causal estimate (*P* < 0.05/number of environmental factors x number of asthma subtypes).

### Statistical Analysis

For the genome-wide association analyses, we used a logistic regression model and assumed an additive genetic model of trait status with genotype dose, fitted using the R package and adjusted for covariates, including age, sex, environmental factor, and PC10s. t-tests were used to compare characteristics between control and asthma cases using R.

We used the TwoSampleMR package for performing Mendelian randomization methods, such as IVW, weighted median, and MR-Egger; the gsmr package for GSMR, the qqman package for drawing Manhattan plot and quantile-quantile (QQ) plots; and the ggplot2 package for drawing odds ratio plots, available in the R stats package, version 3.6.3^3^.

## Results

### Characteristics of Study Population for Asthma Case and Control Subjects

The basic characteristics of the 263,850 UK Biobank participants (35,926 asthma cases and 227,924 controls) in this study are described in Table 1. BMI, obesity, eosinophil parameters, and female frequency were significantly higher in asthma cases than controls, and there was no difference in smoking status between groups.

**TABLE 1:** Characteristics of the asthma cases and controls from the UK Biobank.

	**Control**	**Asthma**
	**(*n* = 227,924)**	**(*n* = 35,926)**
Age (years) *	57.00 ± 7.91	55.86 ± 8.19
Onset age (years)	–	31.07 ± 18.71
Male (%) *	108,409 (47.56%)	15,562 (43.32%)
BMI (kg/m^2^) *	27.26 ± 4.59	28.10 ± 5.28
Obesity (%) *	52,342 (22.96%)	10,468 (29.14%)
Hay fever (%)	–	16,304 (45.38%)
Eosinophil percentage (%) *	2.39 ± 1.67	3.25 ± 2.39
Eosinophil count (10^9^ cells/L) *	0.16 ± 0.12	0.23 ± 0.18
Medicine use (%)	–	16,933 (47.13%)
Smoking status	193,627	30,389
Never smoker	90,354 (46.66%)	14,086 (46.35%)
Previous smoker	80,157 (41.40%)	12,962 (42.65%)
Current smoker	23,116 (11.94%)	3,341 (10.99%)

Obesity, BMI ≥ 30 kg/m^2^; eosinophil percentage, proportion of eosinophils in the leukocytes; and medicine use is used to distinguish between mild and moderate-to-severe asthma.

*P < 7.14E-03 (0.05/7).

### Effects of 69 Environmental Factors on Asthma

**FIGURE 1:**
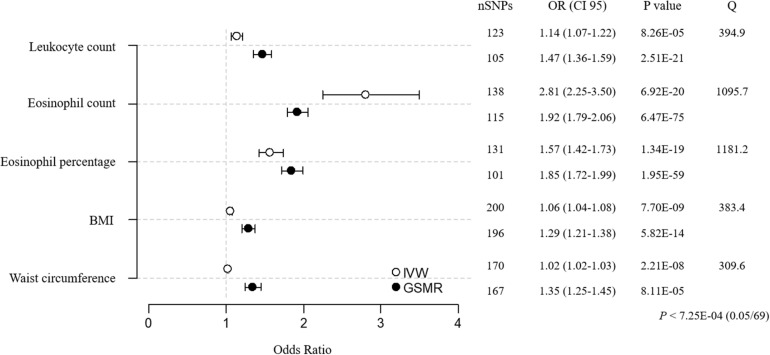
MR estimates for associations between 5 environment factors and asthma. The odds ratio is plotted as dots with the 95% confidence interval. White dots are the IVW method, and black dots are the GSMR method. The *P* value indicates significance when *P* < 7.25E-04 (0.05/69) by Bonferroni correction. The Q value by Cochran’s Q statistic indicates residual heterogeneity in the MR estimates.

Forward MR analyses (environmental factor → asthma) were conducted using IVW, weighted median, and GSMR for 69 factors (Figure 1 and Supplementary Table 4). Factors that were related to white blood cells and anthropometry (leukocyte, eosinophil parameters, BMI, and waist circumference) satisfied the threshold by Bonferroni correction for multiple tests (*P* < 7.25E–04, 0.05/69) in all 3 methods. The intercept *P*-value from the MR-Egger regression suggests that there was relatively balanced pleiotropy (*P* = 0.08 for leukocyte count, *P* = 0.27 for eosinophil count, *P* = 0.47 for eosinophil percentage, *P* = 0.88 for BMI, and *P* = 0.19 for waist circumference). However, the Cochran’s Q values indicated significant residual heterogeneity in all 4 analyses (Q = 394.9, *P*-value = 1.65E–30 for leukocyte count; Q = 1095.7, *P*-value = 3.22E–149 for eosinophil count; Q = 1181.2, *P*-value = 6.41E–169 for eosinophil percentage; Q = 383.4, *P*-value = 8.25E–14 for BMI; and Q = 309.6, *P*-value = 2.46E–10 for waist circumference), which must be improved to determine the true causal effects.

**FIGURE 2:**
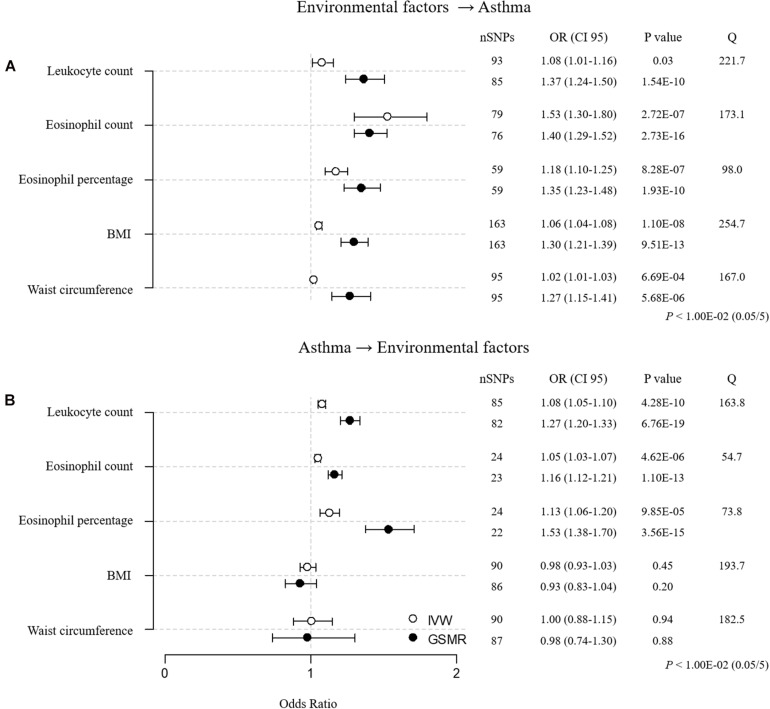
MR estimates for associations between 5 environmental factors and asthma after sensitivity analysis. **(A)** Forward MR: environmental factors → asthma. **(B)** Reverse MR: asthma → environmental factors. The odds ratio is plotted as dots with the 95% confidence interval. White dots are the IVW method, and black dots are the GSMR method. The *P* value indicates significance when *P* < 1.00E-02 (0.05/5) by Bonferroni correction. The Q value by Cochran’s Q statistic indicates residual heterogeneity in the MR estimates.

For the sensitivity analyses, we clumped all 873 environmental factor SNPs (123 for leukocyte count, 138 for eosinophil count, 131 for eosinophil percentage, 200 for BMI, 170 for waist circumference, and 111 for asthma; *r*^2^ < 0.05, 1-Mbp boundary distance; Supplementary Table 15). In addition, we removed environmental factor SNPs that were associated with asthma [*P* < 5.05E–04 (0.05/99) for leukocyte count SNPs, *P* < 5.32E–04 (0.05/94) for eosinophil count SNPs, *P* < 6.17E–04 (0.05/81) for eosinophil percentage SNPs, *P* < 2.99E–04 (0.05/167) for BMI SNPs, and *P* < 5.21E–04 (0.05/96) for waist circumference SNPs] and asthma SNPs that were related to environmental factors [*P* < 5.38E–04 (0.05/93) for asthma SNPs]. RE–analysis of MR using SNPs that were retained after pruning showed that the overall heterogeneity improved, as indicated by the lower Cochran’s Q values (Figure 2A and Supplementary Table 6; Q = 221.7, *P*-value = 1.03E–12 for leukocyte count; Q = 173.1, *P*-value = 3.61E–09 for eosinophil count; Q = 98.0, *P*-value = 8.01E–04 for eosinophil percentage; Q = 254.7, *P*-value = 4.48E–06 for BMI; and Q = 167.0, *P*-value = 5.33E–06 for waist circumference). These additional analyses suggest that eosinophil count, eosinophil percentage, and BMI have causal inferences on asthma, whereas the effects of leukocyte count and waist circumference are not significant, because they failed to reach the threshold for significance [*P* < 1.00E–02 (0.05/5)].

To determine unidirectional causal effects (environmental factor → asthma), we conducted reverse MR analyses (asthma → environmental factor) using asthma SNPs (Figure 2B and Supplementary Table 6). We found that asthma had causal effects on leukocyte count, eosinophil count, and eosinophil percentage, whereas asthma did not causally affect BMI or waist circumference. The reverse MR results indicate that only BMI causally increases the risk of asthma.

### Effects of BMI on the Susceptibility of Asthma Subtypes

Asthma is a highly heterogeneous disease that can be classified by various clinical criteria (Haldar et al., 2008; Zhu et al., 2020). Recent studies indicate that specific asthma subtypes are related to metabolic traits, such as obesity (Jeong et al., 2017). In our study, asthma cases were divided into 4 subsets, based on 2 criteria, combining age of onset and severity: child-onset mild (CM), child-onset moderate-to-severe (CM-S), adult-onset mild (AM), and adult-onset moderate-to-severe (AM-S) (Table 2). The child- and adult-onset moderate-to-severe subgroups had significantly higher BMI, obesity, and eosinophil parameters than their respective mild subgroups (Table 2).

**TABLE 2:** Characteristics of the asthma subtypes and controls from the UK Biobank.

	**Control**	**Child-onset**	**Child-onset**	**Adult-onset**	**Adult-onset**
		**mild**	**moderate-to-severe**	**mild**	**moderate-to-severe**
	**(*n* = 227,924)**	**(*n* = 9,758)**	**(*n* = 1,875)**	**(*n* = 19,415)**	**(*n* = 4,878)**
Age (years)	57.00 ± 7.91	53.86 ± 8.31^a^	55.74 ± 8.45^ab^	56.10 ± 8.01^ad^	58.96 ± 7.42^ace^
Onset age (years)	–	8.62 ± 4.88	8.16 ± 5.37^b^	41.41 ± 12.13^d^	43.62 ± 11.92^ce^
Male (%)	108,409 (47.56%)	5,608 (57.47%)^a^	944 (50.35%)^ab^	7,207 (37.12%)^ad^	1,803 (36.96%)^ace^
BMI (kg/m^2^)	27.26 ± 4.59	27.30 ± 4.78	28.26 ± 5.33^ab^	28.22 ± 5.32^ad^	29.11 ± 5.83^ace^
Obesity (%)	52,342 (22.96%)	2,224 (22.73%)^a^	533 (28.43%)^ab^	5,907 (30.42%)^ad^	1,804 (36.98%)^ace^
Hay fever (%)	–	5,350 (54.68%)	1,099 (58.61%)^b^	7,896 (40.67%)^d^	1,959 (40.16%)^ce^
Eosinophil percentage (%)	2.39 ± 1.67	3.32 ± 2.47^a^	3.74 ± 2.84^ab^	3.13 ± 2.23^ad^	3.39 ± 2.60^ace^
Eosinophil count (10^9^ cells/L)	0.16 ± 0.12	0.23 ± 0.17^a^	0.27 ± 0.23^ab^	0.22 ± 0.17^ad^	0.25 ± 0.20^ace^
Medicine use (%)	–	3,020 (30.86%)	1,875 (100%)	7,160 (36.88%)	4,878 (100%)
Smoking history	193,627	8,081^a^	1,628^a^	16,389^d^	4,291^ace^
Never smoker	90,354 (46.66%)	4,078 (50.46%)	846 (51.97%)	7,503 (45.78%)	1,659 (38.66%)
previous smoker	80,157 (41.40%)	3,076 (38.06%)	615 (37.78%)	7,198 (43.92%)	2,073 (48.31%)
Current smoker	23,116 (11.94%)	927 (11.47%)	167 (10.26%)	1,688 (10.30%)	559 (13.03%)

*Onset age: child-onset <20, adult-onset ≥20; Obesity, BMI ≥30 kg/m^2^; Eosinophil percentage, proportion of eosinophils in the leukocytes; and Medicine use is used to distinguish between mild and moderate-to-severe asthma. ^*a*^*P* < 7.81E–04 (0.05/64) compared with the asthma control group, ^*b*^*P* < 7.81E–04 (0.05/64) vs. the child-onset mild group, ^*c*^*P* < 7.81E–04 (0.05/64) vs. the child-onset moderate-to-severe group, ^*d*^*P* < 7.81E-04 (0.05/64) vs. the child-onset mild group, ^*e*^*P* < 7.81E–04 (0.05/64) vs. the adult-onset mild group.*

**FIGURE 3:**
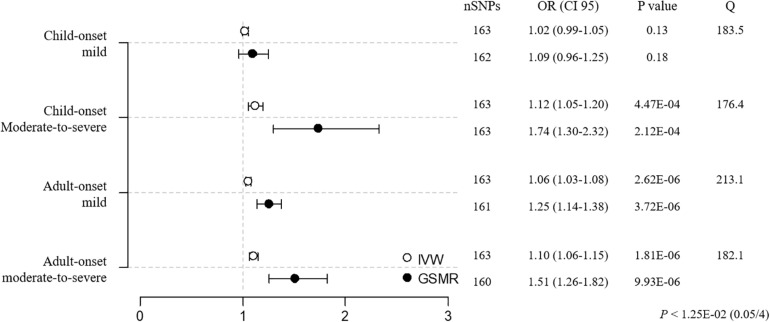
MR estimates of associations between BMI and asthma subtypes. The odds ratio is plotted as dots with the 95% confidence interval. White dots are the IVW method and black dots are the GSMR method. The *P* value indicates significance when *P* < 1.25E-02 (0.05/4) by a Bonferroni correction. The Q value by Cochran’s Q statistic indicates residual heterogeneity in the MR estimates.

Mendelian randomization (BMI → asthma) was performed using 4 asthma subtypes. As a result, the causal impact of BMI on asthma was stronger in moderate-to-severe versus mild cases before sensitivity analysis, with the adult-onset moderate-to-severe subtype the most susceptible to BMI ([CM versus CM-S] IVW OR/CI95 = 1.02/0.99–1.05 versus 1.10/1.04–1.17, weighted median = 1.02/0.98–1.06 vs. 1.09/1.00–1.20, GSMR = 1.09/0.97–1.23 vs. 1.62/1.24–2.12; [AM vs. AM-S] IVW = 1.06/1.03–1.08 vs. 1.12/1.07–1.16, weighted median = 1.02/0.99–1.05 vs. 1.11/1.05–1.17, GSMR = 1.271.17–1.39 vs. 1.67/1.41–1.97; Supplementary Tables 7, 15). Additional sensitivity analyses improved the heterogeneity, as evidenced by the Cochran’s Q values, establishing BMI as having its strongest effect on asthma in the adult-onset moderate-to-severe subgroup ([CM vs. CM-S] IVW OR/CI95 = 1.02/0.99–1.05 vs. 1.12/1.05–1.20, weighted median = 1.01/0.97–1.06 vs. 1.10/1.00–1.22, GSMR = 1.09/0.96–1.25 vs. 1.74/1.30–2.32; [AM vs. AM-S] IVW = 1.06/1.03–1.08 vs. 1.10/1.06–1.15, weighted median = 1.02/0.99–1.06 vs. 1.09/1.03–1.16, GSMR = 1.25/1.14–1.38 vs. 1.51/1.26–1.82; Figure 3 and Supplementary Table 7). The child-onset moderate-to-severe and adult-onset mild subgroups were marginally susceptible to asthma, based on the IVW and GSMR analyses, but not weighted median analysis, suggesting the weak causality of BMI. These findings suggest that the causality of BMI in asthma increases as the severity of asthma rises in child- and adult-onset asthma cases and that the causal effect of BMI is the strongest in the adult-onset moderate-to-severe subgroup.

### Gender-Specific Effect of BMI in Adult Onset Asthma Cases

Observational studies have suggested that BMI causes the development of adult-onset asthma and that the effect of BMI on asthma is greater in female versus male adults (Chen et al., 2002; Guerra et al., 2002; Beuther and Sutherland, 2007), although the gender-specific effect of BMI on asthma is unknown in children (Castro-Rodriguez et al., 2001; Gold et al., 2003). To study the sex-specific causality of BMI, we divided adult-onset asthma cases into 4 subgroups and controls into 2 subgroups by gender: male adult-onset mild (MAM, *n* = 7207), male adult-onset moderate-to-severe (MAM-S, *n* = 1803), female adult-onset mild (FAM, *n* = 12,208), female adult-onset moderate-to-severe (FAM-S, *n* = 3075), male control (MC, *n* = 108,409), and female control (FC, *n* = 119,515). FAM-S and MAM-S patients had higher BMI, obesity, and eosinophil parameters compared with FAM and MAM cases, respectively (Table 3).

**TABLE 3:** Characteristics of the adult-onset asthma cases and controls from the UK Biobank.

**Environment**	**Male**	**Male adult-onset**	**Male adult-onset**	**Female**	**Female adult-onset**	**Female adult-onset**
	**control**	**mild**	**moderate-to-severe**	**control**	**mild**	**moderate-to-severe**
	**(*n* = 108,409)**	**(*n* = 7,207)**	**(*n* = 1,803)**	**(*n* = 119,515)**	**(n = 12,208)**	**(n = 3,075)**
Age (years)	57.21 ± 8.00	56.29 ± 8.19^a^	59.71 ± 7.36^ab^	56.81 ± 7.83	55.99 ± 7.89^a^	58.51 ± 7.42^abc^
Onset age	–	41.74 ± 12.22	44.65 ± 12.03^b^	–	41.21 ± 12.06^d^	43.01 ± 11.81^bc^
BMI (kg/m^2^)	27.80 ± 4.12	28.33 ± 4.50^a^	28.92 ± 4.87^ab^	26.77 ± 4.93	28.16 ± 5.75^a^	29.21 ± 6.33^ab^
Obesity (%)	26,944 (24.85%)	2,156 (29.92%)^a^	625 (34.66%)^ab^	25,398 (21.25%)	3,751 (30.73%)^ad^	1,179 (38.34%)^abc^
Hay fever (%)	–	2,618 (36.33%)	571 (31.67%)^b^	–	5,278 (43.23%)^d^	1,388 (45.14%)^bc^
Eosinophil percentage (%)	2.57 ± 1.75	3.40 ± 2.31^a^	3.64 ± 2.69^a^	2.22 ± 1.57	2.97 ± 2.17^ad^	3.25 ± 2.54^abc^
Eosinophil count (10^9^ cells/L)	0.17 ± 0.13	0.24 ± 0.17^a^	0.27 ± 0.22^ab^	0.15 ± 0.12	0.21 ± 0.16^ad^	0.24 ± 0.19^abc^
Medicine use (%)	–	2,640 (36.63%)	1,803 (100%)	–	4,520 (37.02%)	3,075 (100%)
Smoking history	92,770	6,103	1,600^ab^	100,857	1,0286^ad^	2,691^abc^
Never smoker	37,483 (40.40%)	2,373 (38.88%)	448 (28.00%)	52,871 (52.42%)	5,130 (49.87%)	1,211 (45.00%)
previous smoker	42,282 (45.58%)	3,058 (50.11%)	916 (57.25%)	37,875 (37.55%)	4,140 (40.25%)	1,157 (43.00%)
Current smoker	13,005 (14.02%)	672 (11.01%)	236 (14.75%)	10,111 (10.03%)	1,016 (9.88%)	323 (12.00%)

*Age onset: adult-onset ≥20; Obesity, BMI ≥ 30 kg/m^2^; Eosinophil percentage, proportion of eosinophils in the leukocytes; and Medicine use are used to distinguish between mild and moderate-to-severe asthma. ^*a*^*P* < 8.93E–04 (0.05/56) vs. its respective gender control, ^*b*^*P* < 8.93E–04 (0.05/56) vs. its gender-specific mild group, ^*c*^*P* < 8.93E–04 (0.05/56) vs. the male moderate-to-severe group, ^*d*^*P* < 8.93E-04 (0.05/56) vs the male mild group.*

**FIGURE 4:**
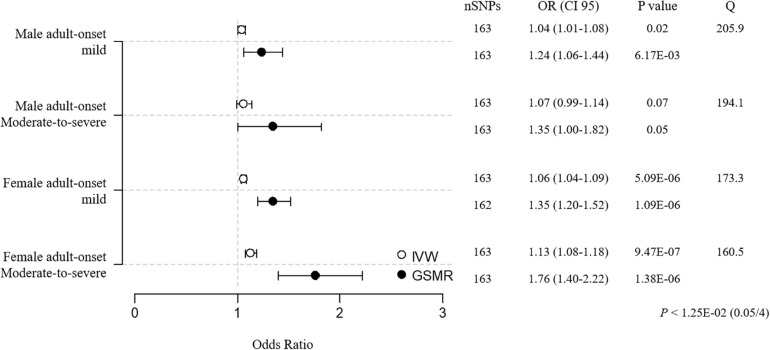
MR estimates of associations between BMI and adult-onset asthma subtypes. The odds ratio is plotted as dots with the 95% confidence interval. White dots are the IVW method, and black dots are the GSMR method. The *P* value indicates significance when *P* < 1.25E-02 (0.05/4) by Bonferroni correction. The Q value by Cochran’s Q statistic indicates residual heterogeneity in the MR estimates.

Prior to the sensitivity analysis, the results in the adult subgroups showed that female adult-onset cases were more susceptible to the causal effect of BMI than male cases ([MAM versus FAM] IVW OR = 1.05 versus 1.06, weighted median OR = 1.05 versus 1.04, GSMR OR = 1.26 versus 1.35; [MAM-S versus FAM-S] IVW OR = 1.08 versus 1.14, weighted median OR = 1.11 versus 1.09, GSMR OR = 1.43 versus 1.83; Supplementary Tables 8, 15). All 3 MR results in the female moderate-to-severe subgroup support the robust inference of BMI on asthma, with the heterogeneity by Cochran’s test implying little pleiotropy. In the sensitivity analyses using clumping of SNPs, the susceptibility to asthma in the female moderate-to-severe cases was marginally significant, because the weighted median regression failed to satisfy the threshold by Bonferroni correction (*P* < 1.25E-02, 0.05/4) (Figure 4 and Supplementary Table 8).

### Effect of BMI on Asthma and Its Subtypes in Two-Sample Mendelian Randomization

To confirm the causal inference of BMI on asthma subtypes without the bias that often occurs in a one-sample setting, we performed two-sample MR using BMI SNPs from another data resource. A total of 149 BMI SNPs were selected from the 158 genome-wide significant SNPs (*P* < 5E-08) that were associated with BMI in a trans-ethnic meta-GWAS of Japanese individuals (the BioBank Japan consortium) and Europeans (the GIANT consortium) (total *N*_max_ = 480,438) (Akiyama et al., 2017). Then, we excluded 2 SNPs that were potentially associated with asthma [*P* < 3.36E-4 (0.05/149)] to mitigate heterogeneity as a sensitivity analysis.

**FIGURE 5:**
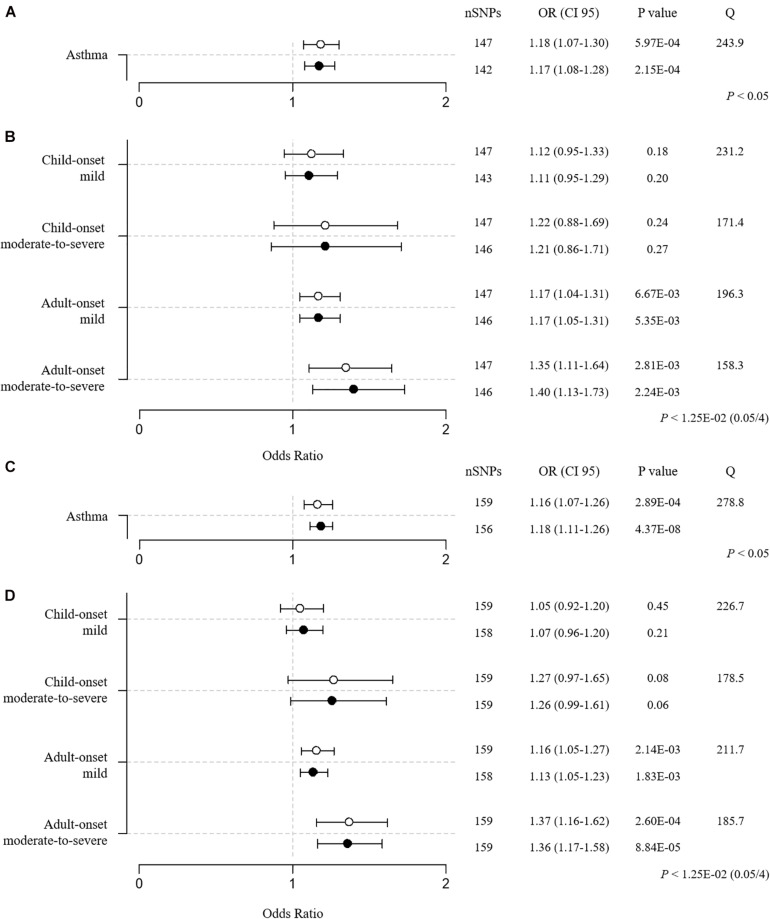
Two-sample MR estimates of BMI → asthma **(A,C)** and BMI → 4 asthma subtypes **(B,D)**. **(A,B)** BMI SNPs were obtained from a meta-GWAS of Japanese (JBB) and European individuals (GIANT). **(C,D)** BMI SNPs were obtained from a GWAS that was performed in only controls (no asthma) from the UK Biobank. The IVW and GSMR methods are indicated by white and black dots, respectively. The Q value by Cochran’s Q statistic indicates residual heterogeneity in the MR estimates.

The MR (BMI → asthma) results of the two-sample setting using 147 BMI SNPs were consistent with the one-sample MR result, implicating BMI as a causal risk factor for asthma [IVW OR = 1.18 (1.07–1.30), *P* = 5.97E–04; weighted median OR = 1.14 (1.01–1.29), *P* = 3.68E–02; GSMR OR = 1.17 (1.08–1.28), *P* = 2.15E–04; Figure 5A and Supplementary Table 9]. Further, in the two-sample MR of the 4 asthma subtypes, the most susceptible was adult-onset moderate-to-severe asthma [IVW OR = 1.35 (1.11–1.64), *P* = 2.81E–03; weighted median OR = 1.52 (1.12–2.06), *P* = 6.76E–03; GSMR OR = 1.40 (1.13–1.73), *P* = 2.24E–03; Figure 5B and Supplementary Table 9], replicating the one-sample MR findings.

To avoid reverse causality or overfitting bias in the one-sample setting, we performed an additional MR analysis using 170 BMI SNPs that were acquired from a genome-wide association analysis with BMI alone in controls (no asthma cases) from the UK Biobank. SNPs were further subjected to clumping by FUMA and exclusion of SNPs that were strand-ambiguous and in the MHC region and SNPs that were potentially associated with asthma (*P* < 3.05E-4 (0.05/164)) as a sensitivity analysis. Ultimately, 159 BMI SNPs were used for the MR (BMI → asthma). The results confirmed BMI as a causal factor for asthma [IVW OR = 1.16 (1.07–1.26), *P* = 2.89E–04; weighted median OR = 1.18 (1.07–1.32), *P* = 1.71E–03; GSMR OR = 1.18 (1.11–1.26), *P* = 4.37E–08; Figure 5C and Supplementary Table 11]. Additional MR analyses of the 4 asthma subtypes confirmed that the adult-onset moderate-to-severe cases were the most susceptible to the development of asthma due to the causal effect of BMI [IVW OR = 1.37 (1.16–1.62), *P* = 2.60E–04; weighted median OR = 1.60 (1.25–2.06), *P* = 2.41E–04; GSMR OR = 1.36 (1.17–1.58), *P* = 8.84E–05; Figure 5D and Supplementary Table 11].

To determine the effect of childhood BMI on asthma in our setting, we extracted 25 childhood BMI SNPs with associated statistical values from a previous study (Vogelezang et al., 2020). These BMI SNPs were subject to the removal of SNPs with strand ambiguity (e.g., A/T and C/G), SNPs in the MHC region (chromosome 6:25-34M), SNPs with no proxy (*r*^2^ > 0.8) in UKB, and SNPs that were associated with asthma (*P* < 2.17E–03, 0.05/23) leaving 21 SNPs. Two-sample MR was performed using these 21 childhood BMI SNPs, and the results are described in Supplementary Table 12. The estimates of childhood BMI in total asthma showed significant effects by IVW (OR/CI95 = 1.11/1.03-1.19, *P*-value = 3.43E–03) and GSMR (OR/CI95 = 1.12/1.04–1.20, *P*-value = 3.43E-03) but not by the weighted median method (OR/CI95 = 1.09/0.99–1.21, *P*-value = 0.07). However, no significant causal effect was found between childhood BMI and the 4 asthma subtypes by IVW, GSMR, and weighted median, consistent with the previous report (Au Yeung et al., 2021). Based on the previous study (Au Yeung et al., 2021) and our result, childhood BMI has a weak causal impact on asthma but not on any specific subtype.

## Discussion

In this study, we used MR to examine the causal relationship between 69 environmental factors and asthma and noted the following: BMI is a causal risk factor for asthma without reverse causation; the effect of BMI on asthma is strongest in the adult-onset moderate-to-severe asthma subgroup; and finally, female subtypes are more prone to asthma due to increased BMI than male subtypes in adults.

Epidemiological and genetic studies that used MR have suggested that BMI is causal factor in asthma (Skaaby et al., 2018; Xu et al., 2019; Sun et al., 2020). In addition, BMI is a risk factor for late-onset asthma (onset age >16) and atopic asthma (Zhu et al., 2020). Our study confirms that BMI is a risk factor for asthma and demonstrates that the causal effect of BMI increases significantly in individuals with child- and adult-onset asthma, exacerbating the asthma. Further, the MR result on the stronger effect of BMI in female subgroups is consistent with observational studies (Chen et al., 2002; Guerra et al., 2002; Beuther and Sutherland, 2007).

In the Epidemiology and Natural History of Asthma: Outcomes and Treatment Regimens (TENOR) study on severe asthma, approximately 57% of individuals with severe asthma were obese, implying a high prevalence of obesity in severe asthma cases compared with an obesity rate of 35% in non-asthma adults in the general United States population (Schatz et al., 2014). In our study, the obesity rate in the adult moderate-to-severe subgroup was significantly higher than in the adult mild subgroup by 7%. Another study found that lung function improved after weight loss in obese patients with asthma, suggesting that greater obesity is related to the severity of asthma (Hakala et al., 2000). There are several potential mechanisms by which BMI is linked to asthma. Obesity has been related to multiple traits of asthma, including eosinophil levels (Kim et al., 2014), lung function (Salome et al., 2010), and allergy (Luo et al., 2013). Studies have suggested that adipokines, such as leptin and adiponectin, are associated with the development and severity of asthma and mediate the exacerbation of asthma through the regulation of eosinophil survival and trafficking (Kim et al., 2014; Zhang et al., 2017; Zheng et al., 2018).

We initially aimed to identify environmental factors that cause the development of asthma. However, of 69 factors, only BMI was identified as a causal influence in asthma. We assume that our study was limited in obtaining the appropriate instruments for certain phenotypes. Environmental data from self-reported questionnaires (e.g., dietary intake, neuroticism, alcohol, smoking, sociodemographic factors, and physical activities) are prone to responder bias (Rask-Andersen et al., 2017). Thus, the presence of an interviewer is recommended to reduce the likelihood of responder bias when obtaining self-reported questionnaire data.

Further, certain anthropometric factors (e.g., fat and non-fat mass) were measured with a less accurate method (bioelectric impedance using a Tanita BC418MA body composition analyzer; UK Biobank) than such techniques as dual-energy x-ray absorptiometry (Speed et al., 2019). Our primary results show that fat mass and percentage, but not fat free mass, are causal factors in asthma by IVW and GSMR (Supplementary Table 4). However, the weighted median method does not support the causal effect of fat mass and percentage. A valid association analysis using precise values can improve the statistics of MR analyses.

Our MR analyses of 69 environmental factors → asthma used only the UK Biobank data as a resource. The one-sample MR setting has several benefits: MR and epidemiological findings can be compared in the same individuals, the validity of causal inferences is unaffected by differences in population characteristics when using 2 samples, and harmonization of genetic variants across datasets is not required (Burgess et al., 2019). However, a limitation of the one-sample analysis is that if the links between genetic instrument and exposure are weak, the causal estimation might suffer from reflection of the confounded associations between exposure and outcome and inflation of false positive (type 1 error) rates (Burgess et al., 2019). Bias in a one-sample analysis with a binary disease outcome can be avoided, such that genetic associations with the exposure are estimated in the controls only; consequently, genetic associations with exposure and outcome will not be correlated (Gharahkhani et al., 2019; Hindy et al., 2019; Wade et al., 2019). We replicated the two-sample MR analyses of BMI → asthma using BMI SNPs from another source of GWAS summary statistics and BMI SNPs from a GWAS only in the control, confirming the one-sample analysis findings.

Although our study stratified asthma by age of onset and sex, concerns remain, because these stratifications were not applied when obtaining genome-wide association statistics on BMI. Genetic studies on BMI have suggested significantly positive genetic correlations between childhood and adult BMI (r_*g*_ = 0.76, *P*-value = 1.45E-112) (Vogelezang et al., 2020) and between male and female BMI (r_*g*_ = 0.879, *P*-value = 5.9E-4) (Yang et al., 2015). Thus, it is likely that depending on the instrument source, BMI might have disparate causal effects on asthma subtypes. Notably, a recent study that used the IVW method confirmed the causal impact of adult BMI on asthma, whereas the possible impact of childhood BMI on the risk of asthma was less clear, mediated predominantly by its relationship with adult BMI, implicating that children with high BMI can reduce their risk of asthma by becoming normal-weight adults. This study was limited, in that there were far fewer childhood BMI SNPs (*N* = 14 and 5) than adult BMI SNPs (*N* = 323 and 115), decreasing the power of the MR estimation (Au Yeung et al., 2021). Our two-sample MR using 25 childhood BMI SNPs (Vogelezang et al., 2020) supports a causal relationship with asthma. However, based on the previous study (Au Yeung et al., 2021) and our result, childhood BMI has a weak causal impact on asthma but not on any specific subtype.

There is much evidence that suggests gender-specific effects of BMI on asthma. Previous epidemiological reports have suggested that the incidence and symptoms of adult asthma are higher and more severe in women than in males (Chen et al., 2003; Zein and Erzurum, 2015). A recent study that performed sex-specific transcriptomics in 5 tissues from asthma patients also showed sexual dimorphism in asthma, including sex-specific dysregulation of genes and signaling pathways (Gautam et al., 2019). Moreover, the effect of BMI on asthma is greater in female than male adults (Chen et al., 2002; Guerra et al., 2002; Beuther and Sutherland, 2007). Consistent with these reports, the asthma cases and M-S asthma subgroups in our study included more female than male adults (Table 3). Further, FAM and FAM-S subtypes had significantly increased obesity compared with MAM and MAM-S subtypes (Table 3). Although we observed that female adult subtypes are more susceptible to the causal effect of BMI than the male groups, the effects did not meet our strict criteria. We speculate that the genetic correlations between male and female asthma subtypes are too high to render any distinctive causal patterns (Supplementary Table 14) compared with correlations between the child- and adult-onset mild and moderate-to-severe subtypes (Supplementary Table 13). For these reasons, it is unlikely that gender-specific BMI instruments have a causal effect on gender-specific asthma subtypes.

In conclusion, our data indicate that elevated BMI levels are causally related to the risk of adult-onset moderate-to-severe asthma. Thus, reducing body weight can help alleviate the susceptibility to moderate-to-severe asthma.

## Data Availability Statement

The datasets presented in this study can be found in online repositories. The names of the repository/repositories and accession number(s) can be found in the article/Supplementary Material.

## Ethics Statement

The studies involving human participants were reviewed and approved by the North West Multicentre Research Ethics Committee, the National Information Governance Board for Health & Social Care, and the Community Health Index Advisory Group. Written informed consent for participation was not required for this study in accordance with the national legislation and the institutional requirements.

## Author Contributions

T-WH, BO, and J-OK designed the study. T-WH analyzed the data and wrote the first draft of the manuscript. J-OK revised the manuscript. BO, JL, HK, and J-OK collected the data and provided technical support. All authors contributed to the interpretation of the results and critical revision of the manuscript for important intellectual content and approved the final version of the manuscript.

## Conflict of Interest

The authors declare that the research was conducted in the absence of any commercial or financial relationships that could be construed as a potential conflict of interest.

## Footnotes

1 https://github.com/Nealelab/UK_Biobank_GWAS

2 http://www.nealelab.is/uk-biobank

3 www.r-project.org

## Abbreviations

AM: adult-onset mild
AM-S: adult-onset moderate-to-severe
BMI: body mass index
BTS: British Thoracic Society
CI: confidence interval
CM: child-onset mild
CM-S: child-onset moderate-to-severe
FC: female control
FAM: female adult-onset mild
FAM-S: female adult-onset moderate-to-severe
GIANT Consortium: The genetic investigation of anthropometric traits consortium
GSMR: generalized summary-data-based mendelian randomization
GWAS: genome-wide association study
HEIDI: heterogeneity in dependent instrument
IVW: inverse-variance weighted
MR: mendelian randomization
MC: male control
MAM: male adult-onset mild
MAM-S: male adult-onset moderate-to-severe
OR: odds ratio
SD: standard deviation
SNP: single-nucleotide polymorphism.
