# Supplementary figures and images for: Association Between Environmental Factors and Asthma Using Mendelian Randomization: Increased Effect of Body Mass Index on Adult-Onset Moderate-to-Severe Asthma Subtypes
Source: Front Genet. 2021 May 20;12:639905. doi: 10.3389/fgene.2021.639905 (PMC8172971; doi:10.3389/fgene.2021.639905)

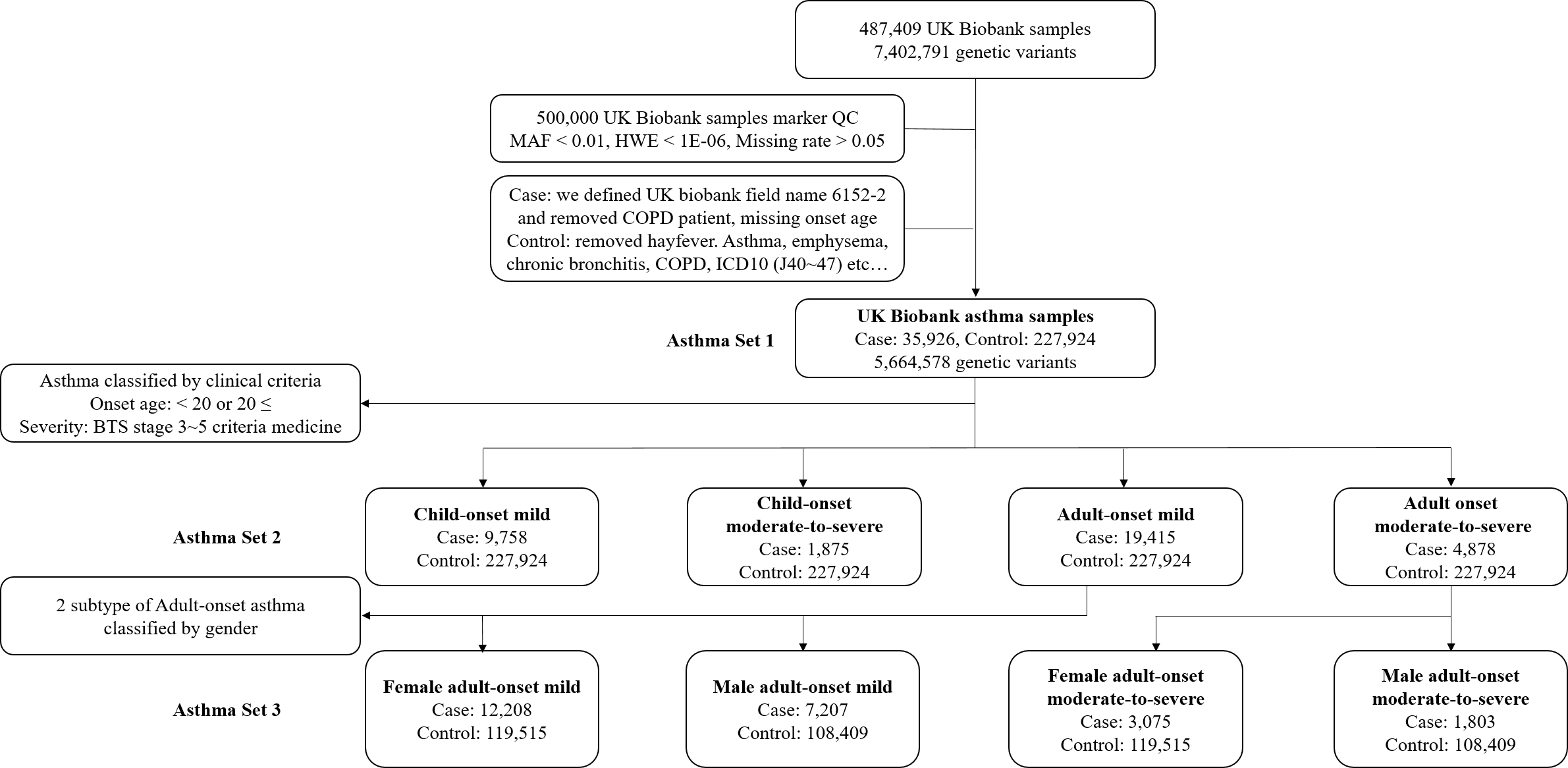

Supplement: Supplementary file 1 [file Image_1.TIF]

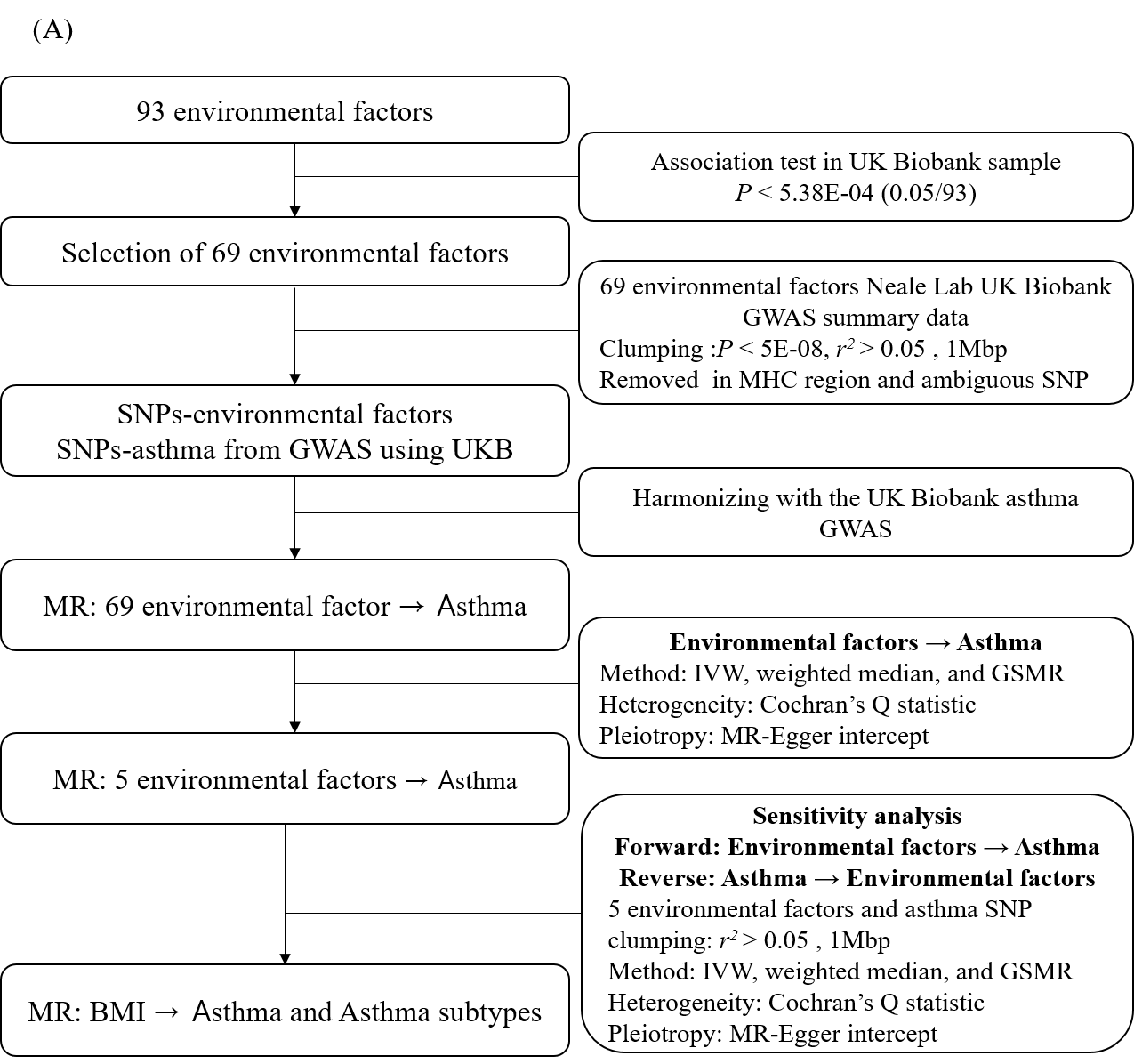

Supplement: Supplementary file 2 [file Image_2.TIF]

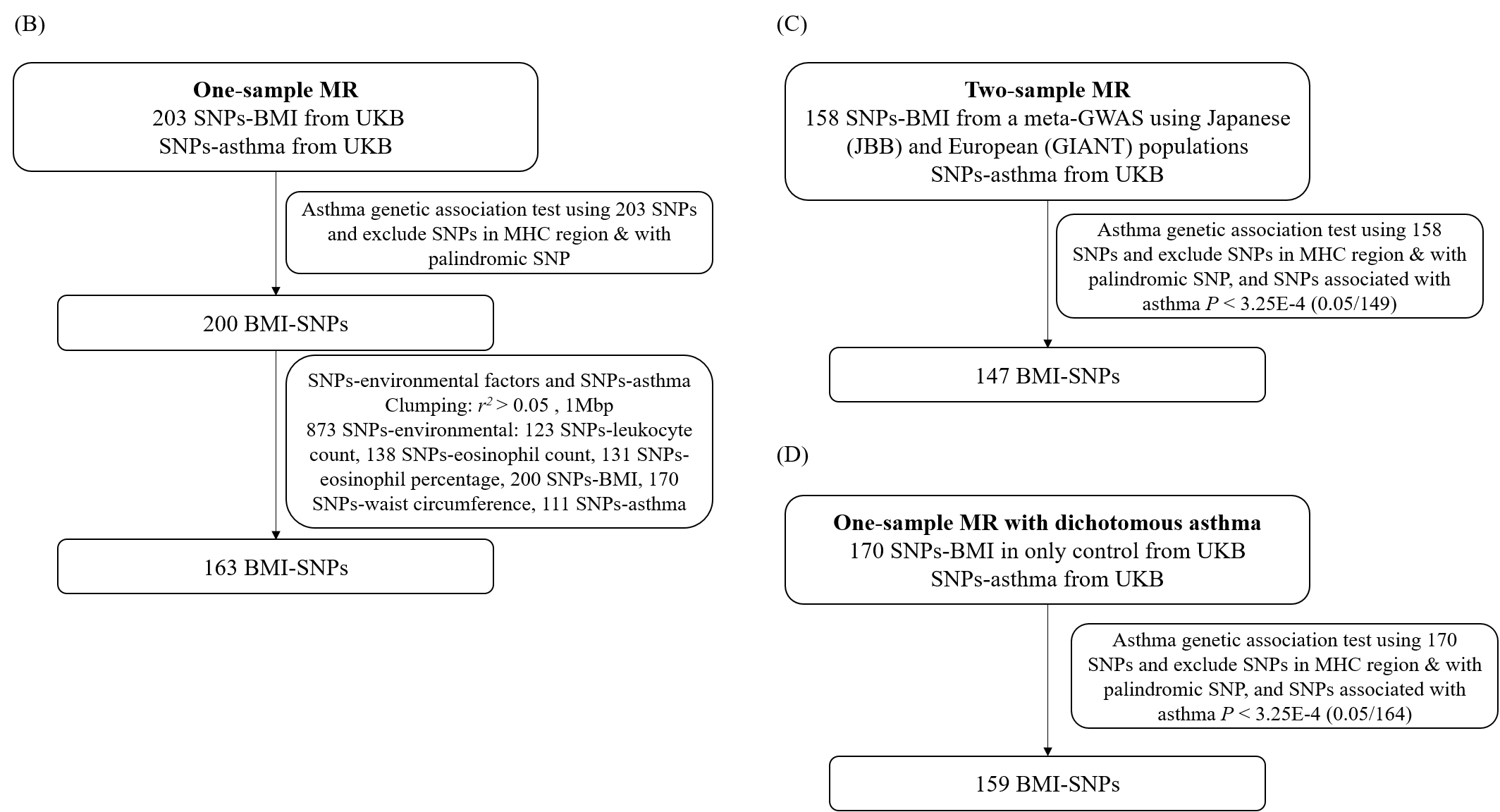

Supplement: Supplementary file 3 [file Image_3.TIF]

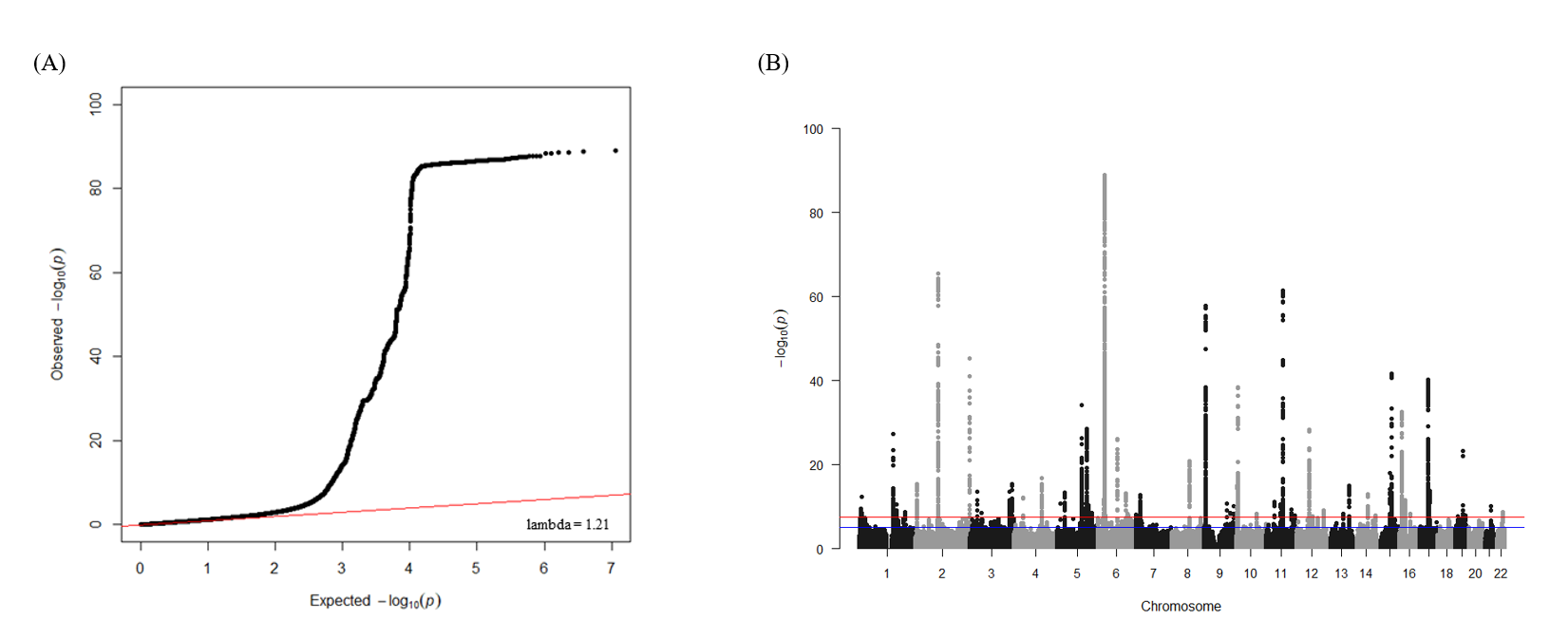

Supplement: Supplementary file 4 [file Image_4.TIF]

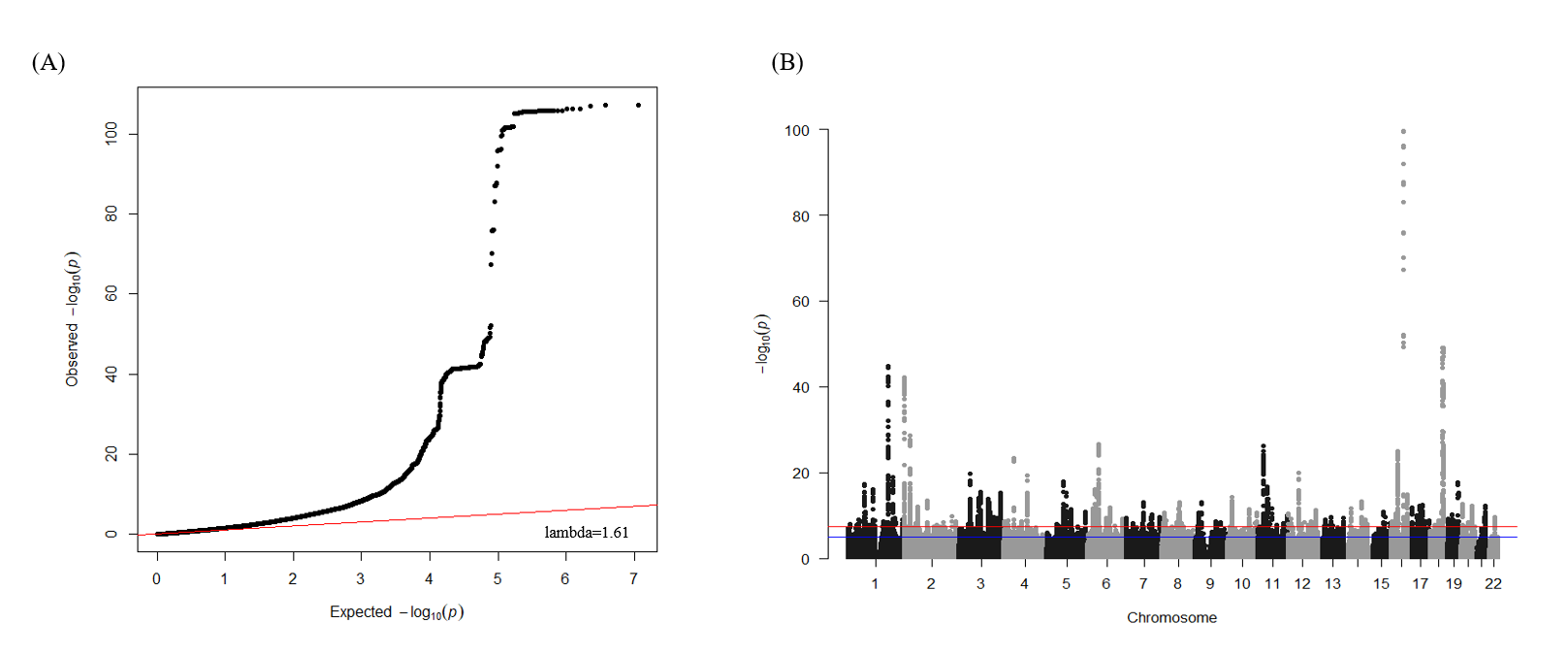

Supplement: Supplementary file 5 [file Image_5.TIF]
