# Supplementary material for: Association Between Environmental Factors and Asthma Using Mendelian Randomization: Increased Effect of Body Mass Index on Adult-Onset Moderate-to-Severe Asthma Subtypes
Source: Front Genet. 2021 May 20;12:639905. doi: 10.3389/fgene.2021.639905 (PMC8172971; doi:10.3389/fgene.2021.639905)
Supplement: Supplementary file 7 [file Table_2.DOCX]

**Supporting materials**

**Study population**

The asthma cases and controls were determined according to the questionnaires of the UK Biobank that the individuals checked as follows:

Asthma cases were defined as the individuals who had checked asthma when asked "Has a doctor ever told you that you have had any of the following conditions?” (data field 6152) The individuals who had not checked in “What was your age when the asthma was first diagnosed?” (data field 3786) or checked in “Has a doctor ever told you that you have had any of the conditions below? - COPD (Chronic Obstructive Pulmonary Disease)” (data field 22130) or who had been the diagnosed COPD age by doctor (data field 22150) were excluded.

Controls were defined as individuals who had not been diagnosed asthma · rhinitis · eczema · allergy · emphysema/chronic bronchitis (data field 6152), also didn’t have data for doctor diagnosed hayfever or allergic rhinitis (data field 22126), age hayfever or allergic rhinitis diagnosed by doctor (data field 22146), doctor diagnosed emphysema (data field 22128), age emphysema diagnosed by doctor (data field 22148), doctor diagnosed chronic bronchitis (data field 22129), age chronic bronchitis diagnosed by doctor (data field 22149), doctor diagnosed COPD (data field 22130), age COPD diagnosed by doctor (data field 22150), ICD10 J40-47 (data field 41202, 41204). Additionally, individuals who had medicated with asthma-related medicines were excluded ([Shrine et al., 2019](#_ENREF_34)).

**Method**

**Genetic correlation**

We used LDSC to estimate LD score intercept and the genetic correlation between asthma subgroup. When using LDSC to estimate genetic correlation and LD score intercept, it is necessary to create an LD score list of causal SNPs (MAF ≥ 0.01). Hence, we derived LD score information as follows: for LDSC, LD score were previously calculated by Bulik-Sullivan et al (https://github.com/bulik/ldsc), based on the European 1000 Genomes database

**Childhood BMI SNPs**

We extracted summary association statistics for the 25 genome-wide significant SNPs (*P* < 5E-08) that had been previously associated with BMI in a meta-genome-wide association study using 61,111 European children with ages of 2-10 years (Vogelezang et al., 2020). From 25 SNPs-BMI, we further removed SNPs with strand-ambiguity (e.g., A/T and C/G) (Tang et al., 2020), SNPs in the MHC region (chromosome 6:25-34M) (Zhu et al., 2020), SNPs with no proxy (*r*^2^ > 0.8) in UKB, and SNPs that were associated with asthma (*P* < 2.17E-03, 0.05/23), leaving 21 SNPs.

**Supplementary Figure**

**Supplementary Figure 1** Study population

**Supplementary Figure 2** MR study design

**Supplementary Figure 3.** The quantile-quantile (QQ) and Manhattan plots of a genome-wide association study on asthma using UK Biobank data. (A) The QQ plot shows the observed versus the expected P-values from the association analyses for asthma in the UK Biobank. The genomic control factor (λ) was 1.21, and the LD Score intercept from LDSC was 1.077, suggesting that any inflation of test statistics is more likely due to polygenicity rather than population structure. (B) European individuals (35,926 asthma cases and 227,924 controls) were used to assess alleles of 5.6 million imputed SNPs. Genome-wide thresholds for significant (*P* = 5E-08) and suggestive (*P* = 5E-06) associations are indicated by the red and blue lines, respectively.

**Supplementary Figure 4** The QQ and Manhattan plots of a genome-wide association study for BMI only in asthma control using UK Biobank data. (A) The genomic control factor (λ) was 1.61, and the LD Score intercept from LDSC was 1.092, suggesting that any inflation of test statistics is more likely due to polygenicity rather than population structure. (B) European individuals (227,924 controls) were used to assess alleles of 5.6 million imputed SNPs. Genome-wide thresholds for significant (*P* = 5E-08) and suggestive (*P* = 5E-06) associations are indicated by the red and blue lines, respectively..
